# Fast nanoscale addressability of nitrogen-vacancy spins via coupling to a dynamic ferromagnetic vortex

**DOI:** 10.1038/ncomms11584

**Published:** 2016-06-14

**Authors:** M. S. Wolf, R. Badea, J. Berezovsky

**Affiliations:** 1 Department of Physics, Case Western Reserve University, Cleveland, Ohio 44106, USA

## Abstract

The core of a ferromagnetic vortex domain creates a strong, localized magnetic field, which can be manipulated on nanosecond timescales, providing a platform for addressing and controlling individual nitrogen-vacancy centre spins in diamond at room temperature, with nanometre-scale resolution. Here, we show that the ferromagnetic vortex can be driven into proximity with a nitrogen-vacancy defect using small applied magnetic fields, inducing significant nitrogen-vacancy spin splitting. We also find that the magnetic field gradient produced by the vortex is sufficient to address spins separated by nanometre-length scales. By applying a microwave-frequency magnetic field, we drive both the vortex and the nitrogen-vacancy spins, resulting in enhanced coherent rotation of the spin state. Finally, we demonstrate that by driving the vortex on fast timescales, sequential addressing and coherent manipulation of spins is possible on ∼100 ns timescales.

## Abstract (web summary)

Nitrogen vacancies in diamond present single spin defects which may be used as nanoscale magnetic sensors. Here, Wolf *et al.* demonstrate how a ferromagnetic vortex in an adjacent micromagnet allows for fast nanoscale addressability of individual defect spins in nanodiamonds.

## Graphical abstract

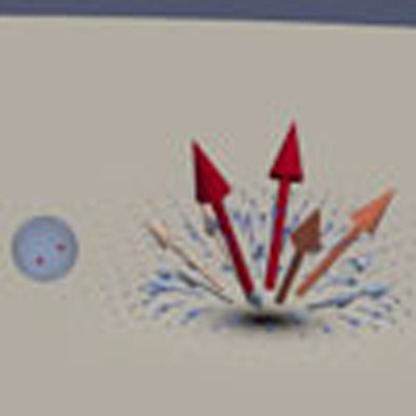

Future spintronic devices will require fast, strong, localized magnetic fields (or effective magnetic fields) in an integrated platform^1^. Ferromagnetic (FM) vortices provide a route towards this requirement, in particular, when coupled to nitrogen-vacancy (NV) centre spins in diamond. NVs are an increasingly attractive candidate for applications in both spin-based sensing^2^,^3^,^4^ and quantum information processing^5^,^6^ because of their long coherence times at room temperature^7^,^8^,^9^ and nanoscale size. But to take advantage of the NV's small size, individual spins must be addressable on nanometre-length scales, for individual manipulation and read-out of the spin state, and to control coupling between proximal spins.

Previous work has studied the possibility of using a magnetic field gradient for spin addressability, although without the necessary combination of nanometre-scale resolution, fast control and potential scalability. By using the static fringe field from a uniformly magnetized micromagnet, spin addressability was demonstrated in coupled gate-defined quantum dots with comparatively large-size scale^10^. Confined spin-wave modes in such micromagnets have been shown to locally couple to NV spins^11^. But for nanoscale NV addressability, larger magnetic field gradients are required than are typically generated by a uniformly magnetized micromagnet. One approach is to use a scanning nanomagnet (that is, a magnetic force microscopy tip) to provide the necessary gradient^12^,^13^. The inverse process, scanning NV magnetometry, in which an NV centre is placed on a scanning probe, has proven to be a powerful technique for revealing the fringe fields from magnetic structures, including vortex domains^14^,^15^,^16^. These scanning probe approaches do not permit fast control. Here, we show that a FM vortex can provide a large local magnetic field and magnetic field gradient for addressing spins, while also being able to rapidly drive the vortex position for coherent control of the NV spin.

## Results

### Characterizing the NV/vortex system

**Figure 1:**
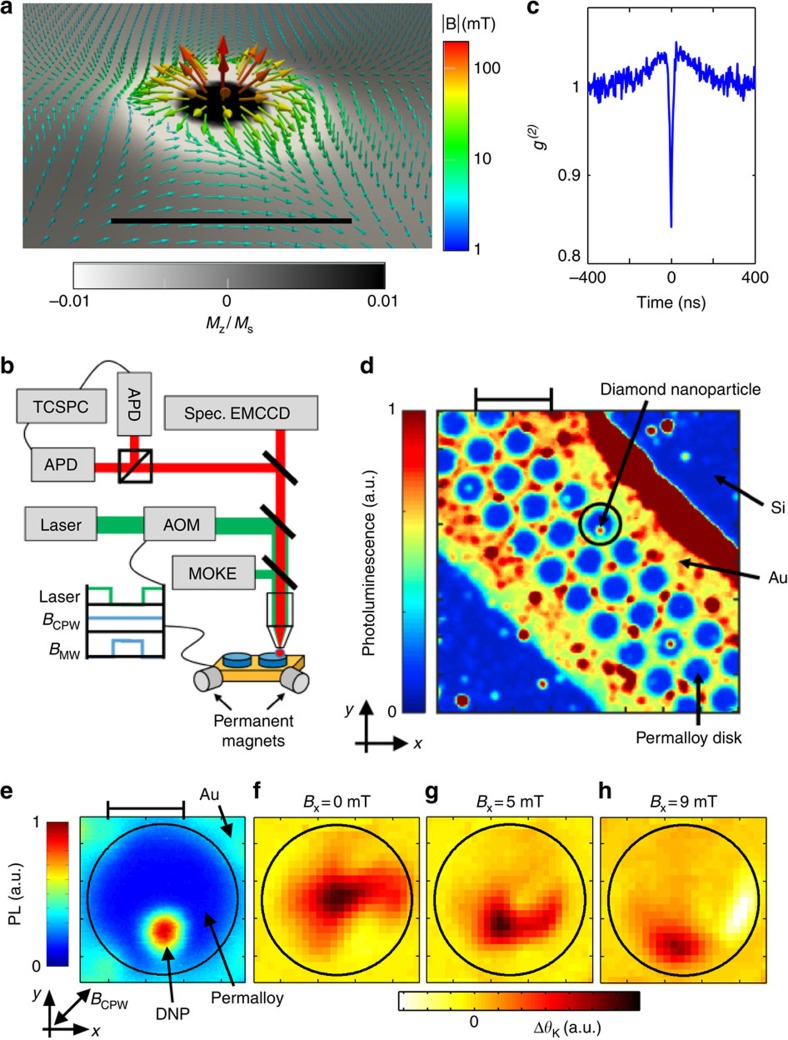
Characterization of the NV/vortex system. (**a**) Micromagnetic simulation near the vortex core with in-plane applied field *B*=3 mT showing normalized out-of-plane magnetization *M*_z_/*M*_s_ (grey scale), and the magnetic field 20 nm above the surface (arrows, logarithmic scale). Scale bar, 100 nm. (**b**) Schematic of the setup. A continuous-wave 532 nm laser is sent through an acousto-optical modulator (AOM) for pulsing. The reflected laser from the sample is sent to a magneto-optical Kerr effect microscopy (MOKE) setup for imaging the vortex core. The PL is directed to either an electron-multiplied charge-coupled device (EMCCD) camera for imaging or sent to a pair of avalanche photodiodes (APDs) connected to a time-correlated single photon counter (TCSPC). (**c**) A *g*^(2)^ measurement of the nanoparticle indicating multiple NV centres. (**d**) PL scan of the sample. Scale bar, 5 μm. (**e**) PL map of the NV/vortex system studied here. The circle indicates the edge of the Permalloy disk, and the double arrow indicates the direction of *B*_CPW_. Scale bar, 1 μm. (**f**–**h**) Differential magneto-optical Kerr effect measurements showing the vortex core positions at different applied fields *B*_x_=0, 5 and 9 mT, respectively.

The core of a ferromagnetic vortex provides a strong, local, controllable magnetic field for spin addressability and control. The ground-state magnetization of a FM microdisk is a single vortex domain with in-plane magnetization circulating around a central core of magnetization normal to the plane^17^,^18^,^19^. A simulation of vortex magnetization near the core, and the resulting fringe field, is shown in Fig. 1a. The grey scale represents the normalized out-of-plane magnetization *M*_z_/*M*_s_, where *M*_s_ is the saturation magnetization. The scale is oversaturated (completely black) at the central vortex core, where *M*_z_≈*M*_s_, in order to show more detail away from the core. The arrows show the magnetic field in a plane 20 nm above the disk surface, with the arrow length and colour showing the field strength on a logarithmic scale. The field is strongest directly above the vortex core, and then falls off approximately as *r*^−3^ with small fields persisting beyond the core due to long-range deformation of the vortex domain.

To demonstrate the vortex-enabled spatial addressability, we need two or more NVs with nanometre-scale separation. Diamond nanoparticles (DNPs) provide a straightforward way to achieve this. We have used DNPs with mean diameter of 25 nm, containing typically one to several NVs. NVs are detected optically in a scanning microscopy setup illustrated in Fig. 1b. Figure 1c shows the two-photon correlation *g*^(2)^ for the particular DNP we study here. The central dip in *g*^(2)^<5/6, indicating that there are roughly 5 or fewer NVs in this DNP. Because the metal substrate also generates some background counts, it is probable that the DNP contains three or four NVs.

NVs and FM vortices are coupled by overcoating a set of Permalloy disks with DNPs. To apply both a microwave (MW) magnetic field with amplitude *B*_MW_ and a rapidly tunable static magnetic field *B*_CPW_, the disks are fabricated atop a gold coplanar waveguide (CPW) as shown in the photoluminescence (PL) scan in Fig. 1d. Diagonally across the scan is a portion (centre) of the CPW. The CPW centre conductor constricts to a 10-μm width for 100 μm at the middle where the Permalloy (Ni_0.81_Fe_0.19_) disks are seen as the array of blue circles. The gold, Permalloy and silicon are distinguishable in the PL scan because of differing background PL counts on these materials. DNPs are dispersed over the entire surface. DNPs generate diffraction limited spots across the image. Their PL intensity is dependent on the number of NVs within the DNP having typically one to several NVs per particle. The specific DNP/vortex system studied is circled in Fig. 1d and a zoomed in PL scan is shown in Fig. 1e. The black circle indicates the edge of a 2-μm-diameter, 40-nm-thick Permalloy disk. The DNP appears as the bright spot in the lower half of the FM disk.

The vortex core position **r**_v_ is controlled by the application of an in-plane magnetic field **B**. To lowest order, |**r**_v_|=*χ*_0_|**B**|, with **r**_v_⊥**B** (refs 20, 21). Differential scanning magneto-optical Kerr effect (MOKE) microscopy is used to image the vortex, and to measure its displacement versus applied field (see the Methods and ref. 22 for details). This technique uses a 15-kHz AC magnetic field with peak amplitude of 1.25 mT to oscillate the vortex position by ≈±100 nm, and measures the resulting change in magnetization. Here, this results in a positive signal Δ*θ*_K_ at the vortex core position, with an amplitude corresponding to the displacement because of the AC field. A static in-plane magnetic field can be applied with arbitrary direction using permanent magnets with automated motion control, and time-dependent fields are generated using the CPW. Figure 1f–h shows differential MOKE images of the vortex, as it is translated by increasing static field *B*_x_. The dark spot shows the location of the vortex core, which translates in the −*y* direction as *B*_x_ is increased. These measurements confirm that a single vortex domain is present and provide a measure of the vortex displacement susceptibility *χ*_0_≈80 nm mT^−1^. The sign of the displacement reveals the sign of the vortex helicity. By comparison to the PL scan in Fig. 1e, we can see that as the field is increased from *B*_x_=0 to 9 mT, the vortex approaches close to the nanoparticle, then continues past.

### Probing the NV/vortex interaction

NV spins are initialized and read-out via the standard optically detected magnetic resonance (ODMR) technique at room temperature^23^,^24^, using the same laser for NV excitation as is used for the MOKE measurements. A typical ODMR spectrum is produced by measuring the PL intensity while sweeping the MW frequency *f*_MW_. Dips in the PL intensity reveal transitions between the *m*_s_=0 to *m*_s_=±1 sublevels of the NV ground state. For a single NV in a magnetic field, we generally expect to see two dips centred around the zero-field splitting *f*_0_=2.87 GHz. See the Methods for more detail on the techniques.

**Figure 2:**
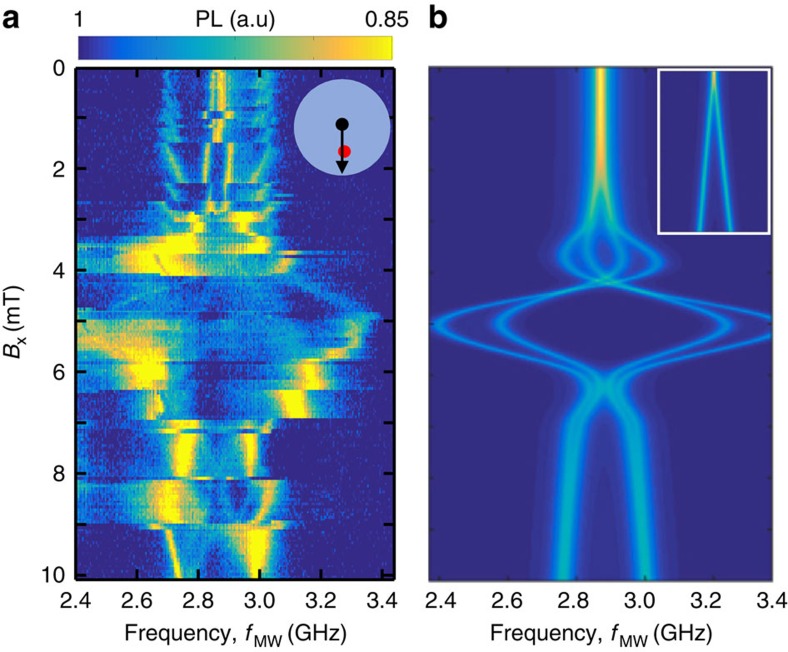
Mapping the NV/vortex interaction. (**a**) Optically detected magnetic resonance (ODMR) spectra versus *B*_x_ of the NVs contained within the DNP. As *B*_x_ increases, the vortex (black dot) follows a path past the NVs (red dot) as illustrated in the inset. (**b**) Simulated ODMR spectra of two NV centres with the same orientation, separated by 10 nm, and 20 nm above the FM disk. Inset shows the same simulation but with no FM disk. The inset axes cover the same range as the main axes.

When the vortex core is brought near an NV (separation *d*_v−NV_<100 nm), the NV spin experiences a significant magnetic field from the vortex. Figure 2a shows ODMR versus *f*_MW_ as the vortex is swept past the NVs by increasing the magnetic field *B*_x_. The path of the vortex **r**_v_(*B*) is shown schematically in the inset (also see Fig. 1f–h for details). The largest splitting occurs around *B*_x_=5 mT, where the MOKE images show that *d*_v−NV_ is small. Here, the transitions to the *m*_s_=+1 and *m*_s_=−1 states are split by ≈900 MHz, corresponding to a projection of ≈16 mT along the NV symmetry axis. The orientations of the symmetry axes of these NVs are unknown, so this sets a lower bound on the actual magnetic field seen by the NV. Given the applied field *B*_x_=5 mT, the vortex must be contributing *B*≥11 mT to the NV spin splitting. As the NV/vortex interaction increases, increased broadening of the resonance is also visible, with linewidths exceeding 100 MHz in some cases. We will show below that this can be explained by power-broadening because of strong vortex-induced amplification of the MW field. A distinct feature of the data in Fig. 2a is that the measured spin splittings do not change continuously with *B*_x_, but instead undergo frequent steps. This is a result of vortex pinning caused by defects in the Permalloy, with steps occurring when the vortex becomes unpinned and jumps to a new equilibrium position^21^.

The strength of the magnetic field gradient from the vortex can be roughly estimated from the slope *γ*_eff_=*δf*/*δB*_x_ of the resonances versus *B*_x_ in Fig. 2a. (Specifically, *γ*_eff_ is related to the gradient in the direction of vortex motion.) Between *B*_x_=4 and 5 mT, we observe the highest *γ*_eff_≈320 MHz mT^−1^. For comparison, the splitting from the applied magnetic field alone is at most *γ*=28 MHz mT^−1^. The observed *γ*_eff_ corresponds to a gradient of the magnetic field projected on the NV axis *B*′=*γ*_eff_/*γχ*_0_≈0.14 mT nm^−1^. For two NVs with the same axis, separated by 10 s of nm, this gradient would cause splitting ∼100 MHz.

The general trends of the resonances versus vortex position can be understood via comparison to simulation. We obtain the fringe field above a FM vortex domain using micromagnetic (object-oriented micromagnetic framework) simulation^25^, as a function of applied field. We then calculate the NV resonances in that field, for NVs in particular locations, in particular orientations. Figure 2b shows the simulated resonances from a pair of NVs, both oriented with polar and azimuthal angles (*φ*, *θ*)=*π*/4. The two NVs are separated by 10 nm, and are located at positions (*x*, *y*, *z*)=(10, −300, 20) nm and (20, −300, 20) nm, relative to the centre of the disk surface. Note that this simulation does not take into account vortex pinning or broadening of the resonances. The broadening may be due to coherent driving of spin-wave modes of the vortex, which is an effect not included in these static simulations. This will be further discussed below. Although the position and orientation of the measured NVs are not precisely known, the simulation captures some general features. The largest splitting is observed when *d*_v−NV_ is near its minimum, around *B*_x_=5 mT. In this region where the vortex is strongly coupled to the NVs, same resonance in different NVs are split ∼100 MHz. When the vortex core is far from the NVs, the resonances tend towards the frequencies expected with no vortex present, shown for comparison in the inset. The experimental data show the same trends, although the experiment displays greater splitting when the vortex core is far from the NVs. This suggests that the fringe field contains higher gradients away from the core, perhaps due to irregularities in the FM film.

### Coherent manipulation of vortex-coupled NV spins

**Figure 3:**
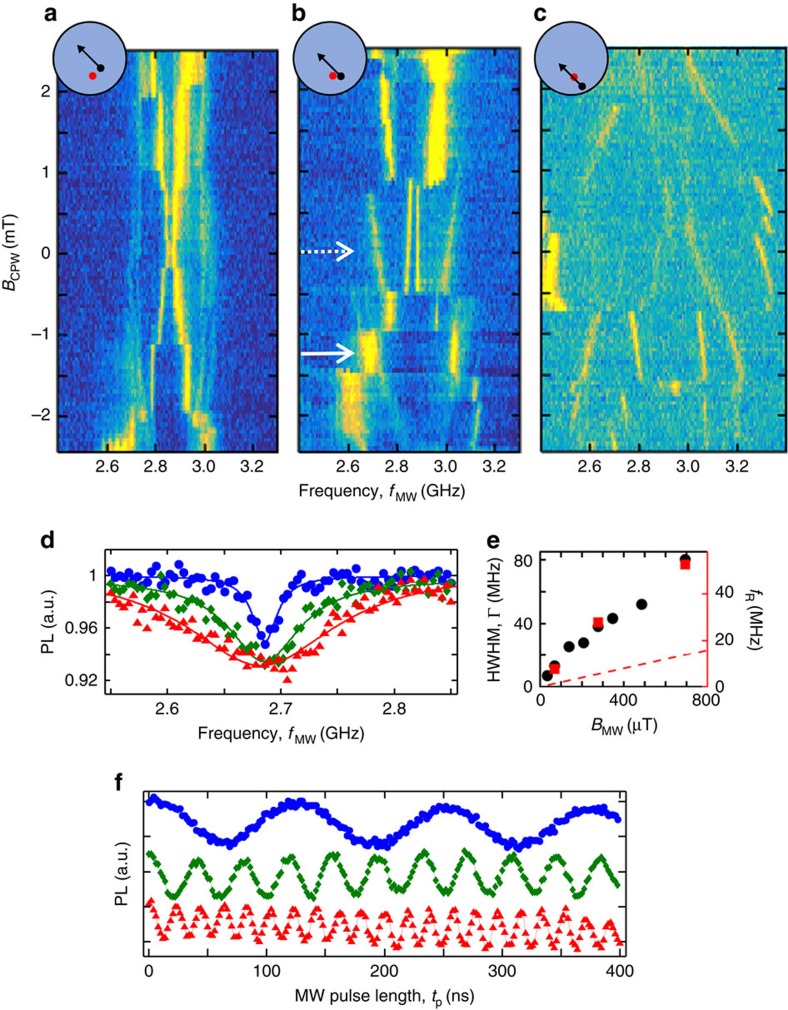
Coherent control of vortex-coupled NV spins. (**a**–**c**) ODMR spectra with static *B*_x_ plus variable *B*_CPW_ translating the vortex along the diagonal paths shown in the inset illustrations. *B*_x_=0, 2, 5 mT, respectively. (**d**) ODMR spectra with Lorentzian fits of the NV spin transition at the field indicated by the solid arrow in **b** with *B*_MW_=70 μT (·), 280 μT (♦) and 690 μT (▴). (**e**) (Left axis, ·) Half-width at half-maximum Γ of the resonance in **d** versus *B*_MW_. (Right axis, ▪) Rabi frequencies *f*_R_ versus *B*_MW_ from the data in **f**. Dashed line indicates upper bound of *f*_R_ with no vortex-induced enhancement. (**f**) Rabi oscillations at the same resonance and the same three *B*_MW_ as in **d**. The oscillations are offset for a clear comparison.

The first step towards fast addressability is to map the NV resonances in response to *B*_CPW_. Sweeping *B*_CPW_ with an additional static field *B*_x_ moves the vortex along the paths illustrated in the insets to Fig. 3a–c. The NV resonances that result from these vortex paths are shown by the corresponding ODMR scans (see the Methods for more detail on the experimental protocol).

The ODMR scans in Fig. 3a–c provide a more detailed look at the NV/vortex coupling. The resonances show a general trend of greater splitting at smaller *d*_v−NV_. The scan in Fig. 3c, closest to the vortex, was taken with reduced *B*_MW_, which reduces the power broadening that obscured the features at small *d*_v−NV_ in Fig. 2a. Sets of resonances with qualitatively different behaviour versus *B*_CPW_ likely arise from NVs with different orientation within the DNP. When lines are split into parallel doublets, this suggests that the transitions of NVs with the same orientation have become addressable. From these maps, we can design a path of **r**_v_ that will sequentially address one or more of the NV transitions.

To demonstrate that the broadening of the resonances is caused by vortex-enhanced power broadening, we measure both the half-width at half maximum Γ and the Rabi frequency *f*_R_ of a spin transition at different *B*_MW_. Figure 3d displays the ODMR spectrum of one of the transitions at the magnetic field indicated by the solid arrow in Fig. 3b. The resonance dip is shown at increasing *B*_MW_ with a corresponding increase in linewidth. A Lorentzian fit yields Γ at these and other *B*_MW_, shown as circles in Fig. 3e (left axis). Γ is roughly linear with *B*_MW_, as expected if linewidth is dominated by MW-field-induced power broadening. We confirm the broadening mechanism by measuring coherent Rabi oscillations of this spin transition (see the Methods for details). Figure 3f shows Rabi oscillations with MW pulses at *f*_MW_=2,688 MHz, and the same *B*_MW_ as used in Fig. 3d. The Rabi frequency *f*_R_, extracted via a Fourier transform, is shown as squares in Fig. 3e (right axis). For comparison, the line 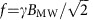 (*γ*=28 MHz mT^−1^) is also shown, which is the maximum possible *f*_R_ for the NVs alone, in the absence of vortex-induced enhancement. Both Γ and *f*_R_ show the same roughly linear trend versus *B*_MW_. In the limit of strong MW-power broadening, the linewidth is expected to be 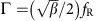, where *β* is the ratio between optical excitation rate and spin initialization rate^26^. From the data in Fig. 3e, we find *β*≈10, consistent with typical values for this ratio. It is possible that this linewidth broadening and Rabi frequency enhancement are caused by coherent MW-induced excitation of spin wave modes in the disk, which in turn drive the spin transitions^11^,^27^.

### Fast NV addressability and coherent manipulation of spins

**Figure 4:**
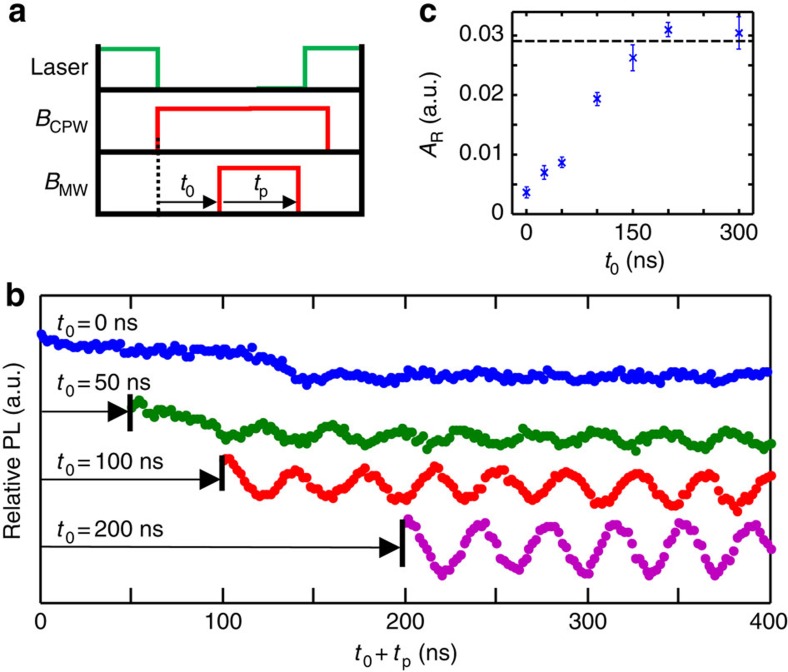
Fast NV addressability and coherent control. (**a**) Pulse timing for NV initialization, and read-out (laser), vortex-enabled addressing (*B*_CPW_) and manipulation *B*_MW_. *t*_0_ is the delay between *B*_CPW_ and *B*_MW_ and *t*_p_ is the *B*_MW_ pulse duration. (**b**) Rabi oscillations following a vortex translation with different *t*_0_. The oscillations are offset for a clear comparison. (**c**) The Rabi amplitude, *A*_R_, versus *t*_0_. Dashed line indicates *A*_R_ with static vortex position. The error bars are determined by the sinusoidal fits.

We now demonstrate the capability to sequentially move the vortex and perform coherent operations on one of the NVs in the DNP. The pulse sequence for this operation is shown in Fig. 4a. The laser serves to first initialize, and finally measure all of the NV spins in the DNP. During the spin initialization, *B*_CPW_=0, so the vortex is in the position indicated by the dashed arrow in Fig. 3b. After the initialization pulse has ended, *B*_CPW_ is stepped to the solid arrow in Fig. 3b. A MW pulse at *f*_MW_=2,688 MHz then coherently rotates the spin. The MW pulse has duration *t*_p_, and is delayed from the *B*_CPW_ pulse by *t*_0_. From the map in Fig. 3b, we see that at the initial vortex position, no NV transitions are resonant with *f*_MW_. After the step in *B*_CPW_, the vortex position undergoes some dynamics, finally relaxing to a position where a spin transition is now resonant with *f*_MW_. The vortex position is reset after the spin read-out (resetting the vortex position before the read-out does not affect the outcome).

The timescale for addressing an NV with the vortex and performing coherent spin rotation is demonstrated by the Rabi oscillations shown in Fig. 4b. The ODMR contrast is plotted versus *t*_0_+*t*_p_ with different *t*_0_. Sinusoidal fits performed at *t*_0_+*t*_p_>200 ns yield the oscillation amplitudes *A*_R_ (Fig. 4c). When the MW pulse begins *t*_0_=200 ns after the *B*_CPW_ step, clear Rabi oscillations are observed, with *A*_R_ equal to the value observed for static vortex position (dashed line in Fig. 4c). As *t*_0_ is reduced, the oscillation contrast is reduced. At *t*_0_≤50 ns, a non-oscillating decay of the signal is visible at *t*_0_+*t*_p_≤100 ns. The observed NV spin behaviour can be understood in terms of the dynamic coupling of the vortex and NV transitions. The relaxation time for gyrotropic vortex dynamics in these structures is ∼50 ns, as measured by time-resolved MOKE (not shown) and in agreement with previous work^28^. Once the gyrotropic precession has fully decayed and the vortex has fully relaxed to its new equilibrium position at *t*_0_≥200 ns, the NV transition is on resonance, and coherent rotation proceeds as usual. During the vortex relaxation, the NV spin experiences a time-dependent spin splitting. If the MW pulse is present during these dynamics, the spin follows a more complex path on the Bloch sphere.

## Discussion

The NV/vortex system opens a path towards large-scale quantum registers, in which the vortex can be repositioned to address a single NV within the register. Specifically, tuning the vortex position to maximally split the spin transitions of one NV will separate those transitions from all other NVs in the register. As shown, the vortex can be repositioned on ∼100 ns timescales, allowing for operations on many NVs within typical coherence times. Technical challenges that remain to be studied and addressed, however, include the effects of pinning on the freedom to position the vortex, and the possible presence of vortex-induced spin dephasing or decoherence.

It is interesting to ask whether the Bloch sphere dynamics that occur when the MW pulse is applied while the vortex is moving are coherent (as in the top curve of Fig. 4b). The time evolution of the spin splitting here is similar to that required for adiabatic passage, suggesting that it may be possible to use the vortex dynamics to generate robust spin rotations. The coupled NV/vortex dynamics will provide a rich system for future study, opening new possibilities for coherent spin control with nanoscale addressability.

## Methods

### Experimental setup

ODMR and MOKE measurements were performed on a home-built scanning microscopy setup at room temperature. A schematic of the setup is shown in Fig. 1b. A continuous wave (CW) 532 nm laser is sent through an acousto-optical modulator (Gooch and Housego) for laser pulsing and directed into an oil immersion objective (Olympus × 100, 1.3 numerical aperture) by a dichroic mirror. The objective focuses the laser onto the sample and both the PL and reflected laser are collected back through the same objective. The reflected laser can be sent to the longitudinal MOKE measurement to map the magnetization component perpendicular to *B*_CPW_ (ref. 22). The magnetization maps are produced by raster scanning the disk while measuring the change in magnetization due to the 15-kHz applied *B*_CPW_ at each pixel. The PL proceeds to an electron-multiplication charge-coupled device camera for imaging or sent to a pair of avalanche photodiodes (PDM-50ct) connected to a time-correlated single-photon counting system (TCSPC, Hydraharp 400).

### Sample preparation

The sample is mounted on a three-dimensional nanopositioning stage (Physik Instrumente P-517.3CL). Permanent magnets are placed on automated translation stages that are positioned to move in the *x*- and *y*-directions indicated in Fig. 1d,e. The static field is measured *in situ* by a two-dimensional hall probe integrated in the sample holder. The sample consists of a 200-nm-thick gold CPW patterned via photo-lithography and thermal evaporation on a silicon substrate. The CPW centre conductor constricts to a 10 μm width for 100 μm at the middle. In this region, Permalloy (Ni_0.81_Fe_0.19_) disks are fabricated via electron beam lithography, electron beam evaporation and liftoff, atop of the gold CPW. Last, DNPs are dispersed over the entire surface.

The DNPs (DiaScence, Quantum Particles), ≈25 nm in diameter, are mixed with a 1.0% solution of polyvinyl alcohol, *M*_W_=85,000–124,000 (Aldrich 363,146) in de-ionized water. Spin coating this solution directly on top of the CPW/Permalloy disks yields a film thickness ≈35 nm.

### Microwave field generation

MW fields are produced by a signal generator (SRS SG386) and amplified (Pasternack PE15A4017). MW signals are either CW, internally modulated at low frequency, or pulsed at higher frequency using I/Q modulation. An arbitrary function generator (Tektronix AFG3052C) and a digital delay pulse generator (SRS DG645) are used to provide signals at direct current (DC) or with square amplitude modulation with fast rise time ∼1 ns. The MW field is combined with DC or square-modulated current with a resistive splitter (MicroCircuits ZFRSC-42-S+) and sent to the CPW. The MW magnetic field (with amplitude referred to as *B*_MW_) produced by the CPW drives both the vortex and the NV spin transitions. The DC or low-frequency-modulated magnetic field (referred to as *B*_CPW_) shifts the NV transition frequencies, and moves the vortex.

### ODMR techniques

There are three types of ODMR scans performed in the main text. The first two methods involve simultaneous spin initialization, measurement and incoherent MW-induced depolarization, whereas the third is a pulsed technique that permits coherent spin control. In all three, a timing scheme is used to provide signal and reference levels, as described below.

During an ODMR scan, the TCSPC continuously measures and time-tags each PL photon count in real time, {*t*_1_, *t*_2_, *t*_3_…}. The TCSPC also places event markers that are synced with the timing of the laser pulses and/or MW field modulation. A time interval is defined as the measurement time, and another interval is defined as the reference time (see below). The markers are then used to determine which counts arrived in which interval.

The generalized measurement described above is employed in three ways. In the first method, NVs are under CW laser excitation with the static magnetic field fixed, while sweeping *f*_MW_, as in Fig. 2a. The MW field amplitude is modulated *B*_MW_(*t*)=*B*_MW_*M*(*t*), where *M*(*t*) is a square wave with one period


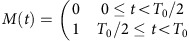


At each *f*_MW_, many modulations occur (for example, *T*_0_=1 ms with measurement time=1 s) and a marker is placed at the beginning of each period of *M*. This allows us to separate the time-tagged PL counts, {*t*_*i*_}, into bins such that the PL signal counts, 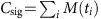, and PL reference counts, 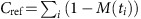. *C*_ref_ reflects the PL count rate when the spins are initialized, with no MW field applied. *C*_sig_ reflects the PL count rate when the spins are initialized, and also acted on by the MW field. The ODMR signal is then defined as *S*=*C*_sig_/*C*_ref_.

The second method is the same as the first, but we also modulate the vortex position via *B*_CPW_ (as in Fig. 3a–c.) This is done to avoid thermal depinning of the vortex. When the vortex is at a position close to a transition between pinning configurations, it can stochastically jump to a new position, in a thermally activated process. Using the first method, the vortex may sit in such a position for many minutes. If a jump occurs during this time, discontinuities can appear in the data in a single *f*_MW_ scan. If the vortex can jump back and forth between two configurations, the scan features can be smeared. To avoid this, we want to reduce the dwell time of the vortex at these unstable positions. Modulating the vortex position back to some reference position essentially resets the vortex. It can still undergo thermal jumps, but if it does, the situation is quickly remedied. Here, both *B*_CPW_(*t*)=*B*_CPW_*M*(*t*) and *B*_MW_(*t*)=*B*_MW_*M*(*t*) are modulated at 1 kHz. In this case, *C*_ref_ pertains to vortex position set by *B*_CPW_=0 with no MW applied, and *C*_sig_ pertains to the vortex with position set by the desired value of *B*_CPW_, with the MW field applied. Because the signal and reference here are measured in different static fields (from both *B*_CPW_ and the vortex field), *C*_ref_ may differ from *C*_sig_ even if no MW field is applied. To account for this, the ODMR signal for each line scan is normalized to have the off-resonant signal, *S*=1.

In the third method, pulsed measurements are carried out, separating the spin initialization, manipulation and measurement in time. We use a measurement protocol with a period of 1.5 μs. The laser is on during the time interval *t*_ON_=(0−1) μs and off during the interval *t*_OFF_=(1−1.5) μs. The measurement time interval (read-out) is defined as *t*_m_=(0−300) ns and the reference time interval is defined as *t*_r_=(700−1,000) ns. *C*_sig_ is then the number of {*t*_*i*_} in the range *t*_m_ and *C*_ref_ is the number of {*t*_*i*_} in the range *t*_r_. The MW field is also pulsed and is on either during *t*_OFF_ (for coherent manipulation, as in Figs 3f and 4b) or during *t*_ON_ (for incoherent depolarization, as in Fig. 3d).

## Additional information

**How to cite this article:** Wolf, M. S. *et al.* Fast nanoscale addressability of nitrogen-vacancy spins via coupling to a dynamic ferromagnetic vortex. *Nat. Commun.* 7:11584 doi: 10.1038/ncomms11584 (2016).
